# *N* ^2^-(4-Meth­oxy­salicyl­idene)arginine hemihydrate

**DOI:** 10.1107/S1600536813019727

**Published:** 2013-07-24

**Authors:** M. Sethuram, G. Bhargavi, M. Dhandapani, G. Amirthaganesan, M. NizamMohideen

**Affiliations:** a Department of Chemistry, Sri Ramakrishna Mission Vidyalaya College of Arts and Science, Coimbatore 641020, Tamil Nadu, India; b School of Chemistry, University of Hyderabad, Hyderabad 500 046, Andhra Pradesh, India; c Department of Physics, The New College (Autonomous), Chennai 600 014, TamilNadu, India

## Abstract

The title compound, C_14_H_20_N_4_O_4_·0.5H_2_O [systematic name: (2*S*)-5-{[amino­(iminium­yl)meth­yl]amino}-2-{[(1*Z*)-4-meth­oxy-2-oxido­benzyl­idene]aza­nium­yl}penta­noate hemihydrate], has been synthesized by the reaction of l-arginine and 4-meth­oxy­salicyl­aldehyde and crystallizes with two independent substituted l-arginine mol­ecules and one water mol­ecule of solvation in the asymmetric unit. Each mol­ecule exists as a zwitterion and adopts a *Z* configuration about the central C=N. The mol­ecular conformation is stabilized by strong intra­molecular N—H⋯O hydrogen bonds that generate *S*(6) and *S*(10) ring motifs. Inter­molecular N—H⋯O and O—H⋯O hydrogen bonds involving also the water mol­ecule and weak inter­molecular C—H⋯O_water_ inter­actions link the mol­ecules into an infinite one-dimensional ribbon structure extending along the *b* axis. The known (2*S*) absolute configuration for l-arginine was invoked. Weak intermolecular C—H⋯π interactions are also present.

## Related literature

For the synthesis of similar compounds, see: Srinivasan *et al.* (1986 ▶); Moutet & Ourari (1997 ▶). For general background on Schiff bases, see: von Konig *et al.* (1982 ▶); Lewis *et al.* (2009 ▶). For hydrogen-bond motifs, see: Bernstein *et al.* (1995 ▶). For related structures, see: Oueslati *et al.* (2007 ▶).
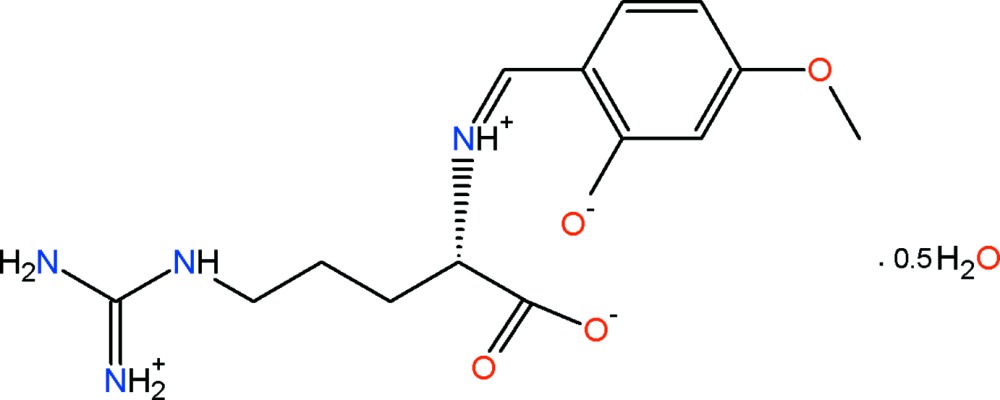


## Experimental

### 

#### Crystal data

C_14_H_20_N_4_O_4_·0.5H_2_O
*M* *_r_* = 317.35
Monoclinic, 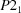

*a* = 10.1828 (11) Å
*b* = 10.3414 (11) Å
*c* = 15.5542 (16) Å
β = 102.688 (2)°
*V* = 1597.9 (3) Å^3^
*Z* = 4
Mo *K*α radiation
μ = 0.10 mm^−1^
*T* = 293 K
0.30 × 0.30 × 0.25 mm

#### Data collection

Bruker Kappa APEXII CCD-detector diffractometer
Absorption correction: multi-scan (*SADABS*; Bruker, 2004 ▶) *T*_min_ = 0.971, *T*_max_ = 0.976
18648 measured reflections
7483 independent reflections
5859 reflections with *I* > 2σ(*I*)
*R*_int_ = 0.036

#### Refinement

*R*[*F* ^2^ > 2σ(*F* ^2^)] = 0.072
*wR*(*F* ^2^) = 0.167
*S* = 1.10
7483 reflections
452 parameters
14 restraints
H atoms treated by a mixture of independent and constrained refinement
Δρ_max_ = 0.25 e Å^−3^
Δρ_min_ = −0.21 e Å^−3^

### 

Data collection: *APEX2* (Bruker, 2004 ▶); cell refinement: *APEX2* and *SAINT* (Bruker, 2004 ▶); data reduction: *SAINT* and *XPREP* (Bruker, 2004 ▶); program(s) used to solve structure: *SHELXS97* (Sheldrick, 2008 ▶); program(s) used to refine structure: *SHELXL97* (Sheldrick, 2008 ▶); molecular graphics: *ORTEP-3 for Windows* (Farrugia, 2012 ▶) and *Mercury* (Macrae *et al.*, 2008 ▶); software used to prepare material for publication: *WinGX* (Farrugia, 2012 ▶) and *PLATON* (Spek, 2009 ▶).

## Supplementary Material

Crystal structure: contains datablock(s) global, I. DOI: 10.1107/S1600536813019727/zs2267sup1.cif

Structure factors: contains datablock(s) I. DOI: 10.1107/S1600536813019727/zs2267Isup2.hkl

Additional supplementary materials:  crystallographic information; 3D view; checkCIF report

## Figures and Tables

**Table 1:** Hydrogen-bond geometry (Å, °)

*D*—H⋯*A*	*D*—H	H⋯*A*	*D*⋯*A*	*D*—H⋯*A*
N1—H1⋯O2	0.89 (1)	1.94 (3)	2.638 (4)	134 (3)
N4—H4*A*⋯O4	0.90 (1)	2.06 (1)	2.935 (4)	165 (3)
N5—H5⋯O6	0.90 (1)	1.90 (3)	2.600 (4)	134 (3)
N8—H8*A*⋯O7	0.90 (1)	2.03 (1)	2.914 (4)	166 (3)
N2—H2⋯O2^i^	0.86	1.92	2.758 (4)	166
N3—H3*A*⋯O3^i^	0.89 (1)	2.58 (3)	3.333 (4)	142 (3)
N3—H3*B*⋯O4^ii^	0.89 (1)	1.93 (1)	2.817 (4)	175 (4)
N4—H4*B*⋯O3^ii^	0.89 (1)	2.03 (1)	2.912 (4)	171 (3)
N6—H6⋯O6^iii^	0.86	1.89	2.705 (4)	158
N7—H7*A*⋯O8^iii^	0.89 (1)	2.50 (3)	3.202 (4)	135 (3)
N7—H7*B*⋯O7^iv^	0.90 (1)	1.91 (1)	2.800 (4)	173 (3)
N8—H8*B*⋯O8^iv^	0.89 (1)	2.05 (2)	2.911 (4)	161 (3)
O1*W*—H2*W*⋯O3^v^	0.94 (1)	1.98 (2)	2.881 (5)	159 (6)
C15—H15*C*⋯O1*W* ^i^	0.96	2.56	3.451 (7)	155
C22—H22⋯O1*W*	0.93	2.53	3.359 (6)	149
C1—H1*C*⋯*Cg*1^vi^	0.96	2.96	3.669 (4)	132
C15—H15*C*⋯*Cg*2^vii^	0.96	2.98	3.762 (5)	139

Symmetry codes: (i) 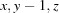; (ii) 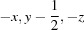; (iii) 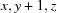; (iv) 
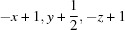
; (v) 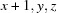; (vi) 
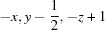
; (vii) 
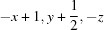
.

**Fig. 1.**
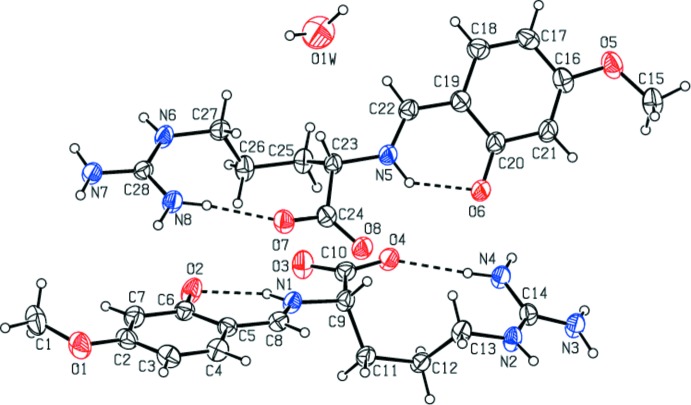
Molecular configuration and atom numbering scheme for the two independent substituted L-arginine molecules and the water molecule of solvation in the asymmetric unit of the title compound. Displacement ellipsoids are drawn at the 30% probability level and intramolecular hydrogen bonds are shown as dashed lines.

**Fig. 2.**
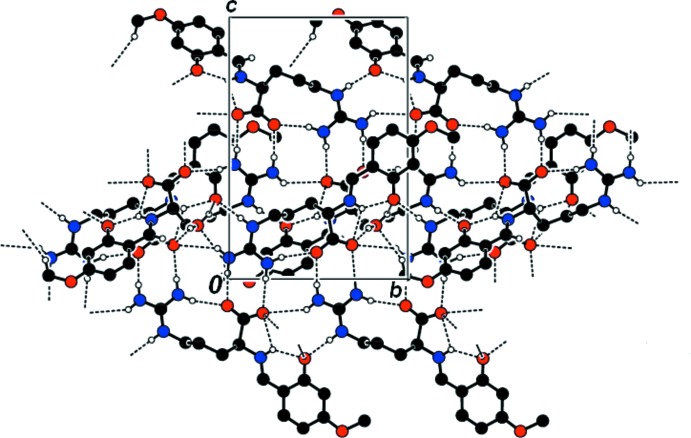
Packing diagram of the title compound viewed down the a axis. Dashed lines indicate intra and intermolecular N—H···O and O—H···O hydrogen bonds and weak C—H···O intermolecular interactions.

## supplementary crystallographic information

### Comment

The Schiff base ligands derived from salicylaldehyde derivatives have been found to be excellent chelating agents for most applications in coordination chemistry such as in catalysis (Srinivasan *et al.*, 1986) and electrocatalysis (Moutet & Ourari, 1997). Schiff bases of the general type *p*-*R*'-C_6_H_4_—CH═N—C_6_H_4_—*R*"-p are well known reagents that find their practical application in various areas, *e.g.* photography (von Konig *et al.*, 1982) and medicinal and pharmaceutical chemistry (Lewis *et al.*, 2009). Here, we report the synthesis of the title compound, C_14_H_20_N_4_O_4_. 0.5H_2_O [systematic name: (2*S*)-5- {[amino(iminio)methyl]amino}-2-{[(1*Z*)-(4-methoxy-2-oxidophenyl) methylene]ammonio}pentanoate hemihydrate] and the structure is reported herein.

This compound crystallizes with two independent substituted *L*-arginine molecules (*A* and *B*), together with one water molecule of solvation in the asymmetric unit (Fig. 1). Each molecule exists as a zwitterion and adopts a *Z* configuration about the central iminium C═N functional group which is coplanar with the adjacent benzene ring. The known (2*S*) absolute configuration for *L*-arginine was invoked for the trivially named chiral centres at C9 and C23. The C—N bond distances of the NH_2_ groups(N3—C14, N4—C14, N7—C28 and N8—C28) are 1.332 (6), 1.319 (6), 1.328 (6) and 1.322 (5) Å, respectively, which is short for a C—N single bond, but still not quite as contracted as one would expect for a fully established C═N. These bond length features are consistent with an imino resonance form as is commonly found for C—N single bonds involving *sp*^2^ hybridized C and N atoms (Oueslati *et al.*, 2007). The bond distances C6—O2, C10—O3, C10—O4, C20—O6, C24—O7 and C24—O8 [1.284 (5), 1.248 (6), 1.239 (5), 1.285 (5), 1.253 (5) and 1.237 (5) Å, respectively], clearly indicate the presence of C═O double bonds, including those also generated through resonance. The H atoms attached to the phenolic groups (O2 and O6) are transferred to the basic centres N1 and N5 respectively, generating the iminium groups. Also, the carboxylic H-atoms on O4 and O7 have been transferred to N4 and N8, respectively, to generate the common amino acid zwitterions.

In both molecules *A* and *B*, all nitrogen H-atoms are involved in hydrogen bonding (Table 1). In each, intramolecular N—H···O hydrogen bonds lead to the formation of a six- and a ten-membered ring motif [*S*(6) and *S*(10), respectively (Bernstein *et al.*, 1995)] (Fig.1). Intermolecular N—H···O and O—H···O hydrogen bonds involving also the water molecule and weak intermolecular C—H···O_water_ interactions link the molecules into an infinite one-dimensional ribbon structure extending along the *b* axis (Fig. 2). Present also are weak intermolecular C—H..π interactions.

### Experimental

*L*-Arginine and 4-methoxy salicylaldehyde (E-Merck- analar grades) were mixed in 1:1 stoichiometric proportions and dissolved in a triply distilled water–ethanol mixture using a mechanical stirrer for about four hours. The raw reaction product was removed by filtration, then re-dissolved in a water–ethanol solvent mixture and kept aside to allow crystal growth at ambient temperature. Bright yellowish crystals formed in 3 days and on removal were recrystallized several times to obtain the crystal specimen used in the X-ray analysis.

### Refinement

The H atoms were positioned geometrically, with methyl C—H distances of 0.96 Å (methylene), 0.93 Å (aromatic) and the N2—H and N6—H distances of 0.86 Å, and were refined as riding on their parent atoms, with *U*_iso_(H) = 1.2–1.5 *U*_eq_ of the parent atom. The remaining N—H atoms and water molecule H atoms were located from a difference Fourier map and refined with distance restraints [N—H = 0.90 (2) and O—H = 0.91 (2) Å] with *U*_iso_(H) = 1.2*U*_eq_(N) and *U*_iso_(H) = 1.5*U*_eq_(O). The known (2*S*) absolute configuration for *L*-arginine was invoked at the trivially numbered chiral centres of the *A* and *B* molecules (C9 and C23, respectively) (Flack parameter: 0.01 (14) for 3448 Friedel pairs).

### Crystal data

**Table:**

C_14_H_20_N_4_O_4_·0.5H_2_O	*F*(000) = 676
*M**_r_* = 317.35	*D*_x_ = 1.319 Mg m^−^^3^
Monoclinic, *P*2_1_	Mo *K*α radiation, λ = 0.71073 Å
Hall symbol: P 2yb	Cell parameters from 3158 reflections
*a* = 10.1828 (11) Å	θ = 2.5–31.2°
*b* = 10.3414 (11) Å	µ = 0.10 mm^−^^1^
*c* = 15.5542 (16) Å	*T* = 293 K
β = 102.688 (2)°	Block, yellow
*V* = 1597.9 (3) Å^3^	0.30 × 0.30 × 0.25 mm
*Z* = 4	

### Data collection

**Table:**

Bruker Kappa APEXII CCD-detector diffractometer	7483 independent reflections
Radiation source: fine-focus sealed tube	5859 reflections with *I* > 2σ(*I*)
Graphite monochromator	*R*_int_ = 0.036
ω and φ scans	θ_max_ = 28.4°, θ_min_ = 2.1°
Absorption correction: multi-scan (*SADABS*; Bruker, 2004)	*h* = −13→13
*T*_min_ = 0.971, *T*_max_ = 0.976	*k* = −13→13
18648 measured reflections	*l* = −20→20

### Refinement

**Table:**

Refinement on *F*^2^	Secondary atom site location: difference Fourier map
Least-squares matrix: full	Hydrogen site location: inferred from neighbouring sites
*R*[*F*^2^ > 2σ(*F*^2^)] = 0.072	H atoms treated by a mixture of independent and constrained refinement
*wR*(*F*^2^) = 0.167	*w* = 1/[σ^2^(*F*_o_^2^) + (0.0716*P*)^2^ + 0.2262*P*] where *P* = (*F*_o_^2^ + 2*F*_c_^2^)/3
*S* = 1.10	(Δ/σ)_max_ < 0.001
7483 reflections	Δρ_max_ = 0.25 e Å^−^^3^
452 parameters	Δρ_min_ = −0.21 e Å^−^^3^
14 restraints	Absolute structure: Flack (1983), 3448 Friedel pairs
Primary atom site location: structure-invariant direct methods	Flack parameter: 0.1 (14)

### Special details

**Table:**

Geometry. All e.s.d.'s (except the e.s.d. in the dihedral angle between two l.s. planes) are estimated using the full covariance matrix. The cell e.s.d.'s are taken into account individually in the estimation of e.s.d.'s in distances, angles and torsion angles; correlations between e.s.d.'s in cell parameters are only used when they are defined by crystal symmetry. An approximate (isotropic) treatment of cell e.s.d.'s is used for estimating e.s.d.'s involving l.s. planes.
Refinement. Refinement of *F*^2^ against ALL reflections. The weighted *R*-factor *wR* and goodness of fit *S* are based on *F*^2^, conventional *R*-factors *R* are based on *F*, with *F* set to zero for negative *F*^2^. The threshold expression of *F*^2^ > σ(*F*^2^) is used only for calculating *R*-factors(gt) *etc*. and is not relevant to the choice of reflections for refinement. *R*-factors based on *F*^2^ are statistically about twice as large as those based on *F*, and *R*- factors based on ALL data will be even larger.

### Fractional atomic coordinates and isotropic or equivalent isotropic displacement parameters (Å^2^)

**Table:**

	*x*	*y*	*z*	*U*_iso_*/*U*_eq_	
C1	0.1564 (4)	1.2584 (4)	0.5422 (3)	0.0774 (13)	
H1A	0.1669	1.3231	0.5874	0.116*	
H1B	0.0660	1.2607	0.5074	0.116*	
H1C	0.2185	1.2750	0.5051	0.116*	
C2	0.1744 (3)	1.0292 (4)	0.5285 (2)	0.0536 (8)	
C3	0.2238 (4)	0.9149 (4)	0.5704 (2)	0.0566 (9)	
H3	0.2572	0.9129	0.6310	0.068*	
C4	0.2224 (3)	0.8064 (4)	0.5214 (2)	0.0515 (8)	
H4	0.2571	0.7303	0.5492	0.062*	
C5	0.1703 (3)	0.8047 (3)	0.4296 (2)	0.0444 (7)	
C6	0.1152 (4)	0.9209 (3)	0.3871 (2)	0.0487 (8)	
C7	0.1196 (4)	1.0328 (4)	0.4389 (2)	0.0559 (9)	
H7	0.0853	1.1101	0.4127	0.067*	
C8	0.1721 (3)	0.6905 (3)	0.3831 (2)	0.0471 (7)	
H8	0.2109	0.6185	0.4146	0.057*	
C9	0.1152 (3)	0.5543 (3)	0.2513 (2)	0.0462 (7)	
H9	0.2055	0.5167	0.2598	0.055*	
C10	0.0613 (4)	0.5805 (3)	0.1526 (2)	0.0499 (8)	
C11	0.0248 (4)	0.4611 (3)	0.2890 (2)	0.0521 (8)	
H11A	0.0645	0.4484	0.3510	0.063*	
H11B	−0.0618	0.5025	0.2849	0.063*	
C12	0.0006 (4)	0.3291 (3)	0.2457 (2)	0.0496 (8)	
H12A	−0.0534	0.2779	0.2773	0.059*	
H12B	−0.0510	0.3401	0.1859	0.059*	
C13	0.1279 (4)	0.2552 (3)	0.2433 (2)	0.0524 (8)	
H13A	0.1827	0.3057	0.2120	0.063*	
H13B	0.1792	0.2420	0.3030	0.063*	
C14	0.0664 (3)	0.1157 (3)	0.1148 (2)	0.0474 (7)	
C15	0.6713 (5)	−0.0130 (4)	0.0132 (3)	0.0757 (12)	
H15A	0.6971	−0.0757	−0.0255	0.114*	
H15B	0.5757	−0.0169	0.0082	0.114*	
H15C	0.7165	−0.0313	0.0728	0.114*	
C16	0.6801 (4)	0.2145 (4)	0.0364 (2)	0.0574 (9)	
C17	0.7369 (4)	0.3301 (4)	0.0165 (2)	0.0575 (9)	
H17	0.7866	0.3324	−0.0270	0.069*	
C18	0.7196 (4)	0.4378 (4)	0.0602 (2)	0.0551 (8)	
H18	0.7587	0.5146	0.0472	0.066*	
C19	0.6423 (3)	0.4371 (3)	0.1264 (2)	0.0494 (8)	
C20	0.5796 (4)	0.3217 (3)	0.1448 (2)	0.0573 (9)	
C21	0.6024 (4)	0.2094 (4)	0.0976 (2)	0.0631 (10)	
H21	0.5639	0.1313	0.1086	0.076*	
C22	0.6243 (3)	0.5520 (3)	0.1693 (2)	0.0480 (7)	
H22	0.6677	0.6254	0.1550	0.058*	
C23	0.5306 (4)	0.6853 (3)	0.2695 (2)	0.0463 (7)	
H23	0.6141	0.7357	0.2800	0.056*	
C24	0.4951 (3)	0.6572 (3)	0.3579 (2)	0.0470 (8)	
C25	0.4207 (4)	0.7613 (3)	0.2061 (2)	0.0532 (8)	
H25A	0.4490	0.7738	0.1510	0.064*	
H25B	0.3392	0.7097	0.1935	0.064*	
C26	0.3882 (4)	0.8927 (3)	0.2403 (2)	0.0550 (9)	
H26A	0.3201	0.9348	0.1956	0.066*	
H26B	0.3509	0.8803	0.2920	0.066*	
C27	0.5096 (4)	0.9796 (3)	0.2639 (2)	0.0582 (9)	
H27A	0.5780	0.9366	0.3078	0.070*	
H27B	0.5460	0.9925	0.2119	0.070*	
C28	0.4773 (3)	1.1249 (3)	0.3797 (2)	0.0456 (7)	
N1	0.1241 (3)	0.6759 (3)	0.29926 (18)	0.0476 (6)	
N2	0.0979 (3)	0.1312 (3)	0.20033 (17)	0.0545 (7)	
H2	0.1009	0.0637	0.2330	0.065*	
N3	0.0323 (4)	−0.0009 (3)	0.0810 (2)	0.0585 (8)	
N4	0.0709 (3)	0.2127 (3)	0.06010 (18)	0.0525 (7)	
N5	0.5525 (3)	0.5643 (3)	0.22704 (17)	0.0482 (6)	
N6	0.4817 (4)	1.1051 (3)	0.29785 (19)	0.0597 (8)	
H6	0.4674	1.1695	0.2620	0.072*	
N7	0.4545 (3)	1.2428 (3)	0.40673 (19)	0.0524 (7)	
N8	0.4954 (4)	1.0294 (3)	0.43757 (19)	0.0575 (8)	
O1	0.1831 (3)	1.1333 (3)	0.58193 (18)	0.0720 (8)	
O2	0.0628 (3)	0.9232 (2)	0.30397 (14)	0.0621 (7)	
O3	0.0088 (3)	0.6877 (3)	0.13073 (16)	0.0692 (8)	
O4	0.0710 (3)	0.4893 (2)	0.10247 (15)	0.0584 (6)	
O5	0.7073 (3)	0.1124 (3)	−0.01041 (17)	0.0733 (8)	
O6	0.5054 (3)	0.3186 (3)	0.20185 (19)	0.0779 (9)	
O7	0.5093 (3)	0.7516 (3)	0.40963 (15)	0.0587 (6)	
O8	0.4554 (3)	0.5477 (2)	0.37124 (16)	0.0586 (6)	
O1W	0.8113 (4)	0.8224 (5)	0.2043 (3)	0.1180 (13)	
H1W	0.834 (6)	0.892 (5)	0.244 (4)	0.177*	
H2W	0.889 (4)	0.783 (6)	0.194 (5)	0.177*	
H4A	0.081 (3)	0.2932 (15)	0.082 (2)	0.044 (9)*	
H7A	0.459 (4)	1.306 (3)	0.3681 (19)	0.058 (11)*	
H4B	0.056 (4)	0.205 (4)	0.0015 (7)	0.060 (11)*	
H7B	0.461 (3)	1.251 (3)	0.4649 (8)	0.047 (9)*	
H8A	0.492 (4)	0.9466 (15)	0.420 (2)	0.055 (10)*	
H8B	0.492 (4)	1.045 (4)	0.4935 (10)	0.056 (10)*	
H3A	0.021 (4)	−0.064 (3)	0.118 (2)	0.065 (12)*	
H3B	0.003 (3)	−0.009 (4)	0.0228 (8)	0.054 (10)*	
H5	0.513 (3)	0.493 (2)	0.241 (2)	0.053 (10)*	
H1	0.083 (3)	0.744 (2)	0.271 (2)	0.049 (9)*	

### Atomic displacement parameters (Å^2^)

**Table:**

	*U*^11^	*U*^22^	*U*^33^	*U*^12^	*U*^13^	*U*^23^
C1	0.063 (2)	0.064 (3)	0.102 (3)	0.002 (2)	0.011 (2)	−0.035 (3)
C2	0.0496 (19)	0.061 (2)	0.0531 (19)	−0.0094 (17)	0.0164 (15)	−0.0168 (17)
C3	0.055 (2)	0.069 (2)	0.0425 (17)	−0.0028 (18)	0.0048 (15)	−0.0087 (18)
C4	0.0464 (18)	0.055 (2)	0.0472 (18)	−0.0038 (15)	−0.0021 (14)	−0.0025 (16)
C5	0.0464 (17)	0.0452 (17)	0.0428 (16)	−0.0023 (14)	0.0126 (14)	−0.0029 (14)
C6	0.0536 (19)	0.0472 (19)	0.0475 (18)	−0.0096 (15)	0.0158 (15)	−0.0057 (15)
C7	0.065 (2)	0.0477 (19)	0.056 (2)	−0.0052 (17)	0.0158 (17)	−0.0047 (16)
C8	0.0481 (18)	0.0470 (17)	0.0459 (17)	−0.0023 (15)	0.0097 (14)	0.0040 (15)
C9	0.0590 (19)	0.0395 (17)	0.0390 (16)	−0.0023 (15)	0.0085 (14)	−0.0018 (13)
C10	0.062 (2)	0.048 (2)	0.0410 (17)	−0.0091 (16)	0.0149 (15)	−0.0017 (15)
C11	0.075 (2)	0.0420 (18)	0.0416 (17)	−0.0030 (16)	0.0188 (16)	0.0003 (14)
C12	0.067 (2)	0.0421 (17)	0.0416 (16)	−0.0115 (16)	0.0158 (15)	−0.0018 (14)
C13	0.072 (2)	0.0427 (17)	0.0383 (16)	−0.0027 (17)	0.0039 (15)	−0.0006 (14)
C14	0.0542 (19)	0.0400 (17)	0.0479 (18)	0.0086 (15)	0.0110 (15)	−0.0007 (15)
C15	0.092 (3)	0.056 (2)	0.077 (3)	0.007 (2)	0.016 (2)	−0.017 (2)
C16	0.074 (2)	0.057 (2)	0.0395 (17)	0.0196 (18)	0.0091 (16)	0.0011 (15)
C17	0.062 (2)	0.070 (2)	0.0436 (18)	0.0046 (19)	0.0186 (16)	−0.0002 (17)
C18	0.058 (2)	0.058 (2)	0.0509 (19)	−0.0003 (17)	0.0150 (16)	0.0013 (17)
C19	0.0538 (19)	0.0484 (19)	0.0457 (17)	0.0080 (15)	0.0103 (14)	0.0053 (15)
C20	0.085 (3)	0.0418 (18)	0.0497 (19)	0.0124 (18)	0.0257 (18)	0.0095 (16)
C21	0.098 (3)	0.0403 (18)	0.057 (2)	0.0063 (19)	0.030 (2)	0.0084 (16)
C22	0.0561 (19)	0.0436 (17)	0.0435 (16)	0.0008 (15)	0.0090 (14)	0.0040 (14)
C23	0.062 (2)	0.0387 (16)	0.0384 (16)	0.0033 (15)	0.0125 (14)	0.0014 (13)
C24	0.0542 (19)	0.0458 (19)	0.0395 (17)	0.0143 (15)	0.0068 (14)	0.0091 (14)
C25	0.071 (2)	0.0432 (17)	0.0397 (17)	0.0071 (17)	0.0006 (15)	0.0000 (14)
C26	0.077 (2)	0.0447 (18)	0.0380 (17)	0.0116 (17)	0.0017 (16)	0.0011 (14)
C27	0.091 (3)	0.0456 (18)	0.0443 (18)	0.0001 (19)	0.0285 (18)	0.0043 (15)
C28	0.0564 (19)	0.0407 (17)	0.0414 (17)	−0.0062 (15)	0.0144 (14)	0.0009 (14)
N1	0.0623 (18)	0.0359 (14)	0.0433 (15)	0.0001 (13)	0.0085 (13)	0.0033 (12)
N2	0.086 (2)	0.0386 (14)	0.0359 (14)	0.0072 (14)	0.0062 (14)	0.0056 (12)
N3	0.090 (2)	0.0406 (16)	0.0440 (17)	−0.0011 (15)	0.0131 (16)	−0.0001 (14)
N4	0.082 (2)	0.0396 (15)	0.0363 (14)	−0.0004 (14)	0.0131 (14)	0.0004 (12)
N5	0.0685 (18)	0.0377 (14)	0.0424 (14)	0.0028 (13)	0.0205 (13)	0.0037 (11)
N6	0.104 (2)	0.0340 (14)	0.0457 (16)	0.0022 (15)	0.0258 (16)	0.0065 (12)
N7	0.0753 (19)	0.0424 (16)	0.0406 (15)	−0.0044 (14)	0.0153 (14)	−0.0013 (13)
N8	0.093 (2)	0.0435 (17)	0.0388 (15)	−0.0007 (16)	0.0215 (15)	0.0025 (13)
O1	0.083 (2)	0.0680 (18)	0.0646 (16)	−0.0093 (15)	0.0160 (14)	−0.0276 (14)
O2	0.0966 (19)	0.0432 (13)	0.0405 (13)	−0.0050 (13)	0.0020 (12)	0.0025 (11)
O3	0.108 (2)	0.0544 (16)	0.0412 (13)	0.0161 (15)	0.0071 (13)	0.0039 (12)
O4	0.0903 (19)	0.0466 (13)	0.0413 (12)	−0.0059 (13)	0.0210 (12)	−0.0030 (11)
O5	0.107 (2)	0.0646 (17)	0.0525 (15)	0.0202 (16)	0.0257 (15)	−0.0042 (13)
O6	0.133 (3)	0.0394 (13)	0.0811 (19)	0.0060 (16)	0.0668 (19)	0.0087 (14)
O7	0.0839 (18)	0.0523 (14)	0.0415 (12)	0.0089 (13)	0.0170 (12)	0.0003 (11)
O8	0.0798 (17)	0.0455 (14)	0.0527 (14)	0.0036 (13)	0.0189 (12)	0.0084 (11)
O1W	0.097 (3)	0.116 (3)	0.136 (4)	0.000 (2)	0.015 (2)	0.010 (3)

### Geometric parameters (Å, º)

**Table:**

C1—O1	1.434 (5)	C17—C18	1.336 (5)
C1—H1A	0.9600	C17—H17	0.9300
C1—H1B	0.9600	C18—C19	1.428 (5)
C1—H1C	0.9600	C18—H18	0.9300
C2—O1	1.351 (4)	C19—C22	1.394 (5)
C2—C7	1.383 (5)	C19—C20	1.412 (5)
C2—C3	1.390 (5)	C20—O6	1.286 (4)
C3—C4	1.355 (5)	C20—C21	1.420 (5)
C3—H3	0.9300	C21—H21	0.9300
C4—C5	1.409 (4)	C22—N5	1.282 (4)
C4—H4	0.9300	C22—H22	0.9300
C5—C8	1.388 (5)	C23—N5	1.454 (4)
C5—C6	1.426 (5)	C23—C24	1.525 (4)
C6—O2	1.286 (4)	C23—C25	1.535 (5)
C6—C7	1.405 (5)	C23—H23	0.9800
C7—H7	0.9300	C24—O8	1.235 (4)
C8—N1	1.297 (4)	C24—O7	1.253 (4)
C8—H8	0.9300	C25—C26	1.523 (5)
C9—N1	1.455 (4)	C25—H25A	0.9700
C9—C11	1.535 (5)	C25—H25B	0.9700
C9—C10	1.537 (4)	C26—C27	1.507 (6)
C9—H9	0.9800	C26—H26A	0.9700
C10—O4	1.241 (4)	C26—H26B	0.9700
C10—O3	1.245 (4)	C27—N6	1.452 (4)
C11—C12	1.518 (5)	C27—H27A	0.9700
C11—H11A	0.9700	C27—H27B	0.9700
C11—H11B	0.9700	C28—N6	1.300 (4)
C12—C13	1.512 (5)	C28—N8	1.321 (4)
C12—H12A	0.9700	C28—N7	1.326 (4)
C12—H12B	0.9700	N1—H1	0.891 (10)
C13—N2	1.448 (4)	N2—H2	0.8600
C13—H13A	0.9700	N3—H3A	0.894 (10)
C13—H13B	0.9700	N3—H3B	0.892 (10)
C14—N2	1.309 (4)	N4—H4A	0.898 (10)
C14—N4	1.323 (4)	N4—H4B	0.894 (10)
C14—N3	1.330 (4)	N5—H5	0.896 (10)
C15—O5	1.418 (5)	N6—H6	0.8600
C15—H15A	0.9600	N7—H7A	0.893 (10)
C15—H15B	0.9600	N7—H7B	0.896 (10)
C15—H15C	0.9600	N8—H8A	0.898 (10)
C16—O5	1.346 (4)	N8—H8B	0.893 (10)
C16—C21	1.366 (5)	O1W—H1W	0.940 (10)
C16—C17	1.393 (6)	O1W—H2W	0.937 (10)
			
O1—C1—H1A	109.5	C19—C18—H18	119.4
O1—C1—H1B	109.5	C22—C19—C20	120.7 (3)
H1A—C1—H1B	109.5	C22—C19—C18	119.5 (3)
O1—C1—H1C	109.5	C20—C19—C18	119.7 (3)
H1A—C1—H1C	109.5	O6—C20—C19	121.4 (3)
H1B—C1—H1C	109.5	O6—C20—C21	121.5 (3)
O1—C2—C7	123.8 (4)	C19—C20—C21	117.1 (3)
O1—C2—C3	114.9 (3)	C16—C21—C20	120.9 (4)
C7—C2—C3	121.3 (3)	C16—C21—H21	119.5
C4—C3—C2	118.8 (3)	C20—C21—H21	119.5
C4—C3—H3	120.6	N5—C22—C19	125.1 (3)
C2—C3—H3	120.6	N5—C22—H22	117.5
C3—C4—C5	122.4 (3)	C19—C22—H22	117.5
C3—C4—H4	118.8	N5—C23—C24	109.6 (3)
C5—C4—H4	118.8	N5—C23—C25	108.2 (3)
C8—C5—C4	119.6 (3)	C24—C23—C25	113.1 (3)
C8—C5—C6	121.6 (3)	N5—C23—H23	108.6
C4—C5—C6	118.8 (3)	C24—C23—H23	108.6
O2—C6—C7	121.0 (3)	C25—C23—H23	108.6
O2—C6—C5	121.1 (3)	O8—C24—O7	127.0 (3)
C7—C6—C5	117.9 (3)	O8—C24—C23	118.6 (3)
C2—C7—C6	120.8 (4)	O7—C24—C23	114.5 (3)
C2—C7—H7	119.6	C26—C25—C23	114.6 (3)
C6—C7—H7	119.6	C26—C25—H25A	108.6
N1—C8—C5	125.3 (3)	C23—C25—H25A	108.6
N1—C8—H8	117.3	C26—C25—H25B	108.6
C5—C8—H8	117.3	C23—C25—H25B	108.6
N1—C9—C11	108.8 (3)	H25A—C25—H25B	107.6
N1—C9—C10	109.1 (3)	C27—C26—C25	112.8 (3)
C11—C9—C10	112.3 (3)	C27—C26—H26A	109.0
N1—C9—H9	108.8	C25—C26—H26A	109.0
C11—C9—H9	108.8	C27—C26—H26B	109.0
C10—C9—H9	108.8	C25—C26—H26B	109.0
O4—C10—O3	126.4 (3)	H26A—C26—H26B	107.8
O4—C10—C9	115.6 (3)	N6—C27—C26	114.0 (3)
O3—C10—C9	117.9 (3)	N6—C27—H27A	108.8
C12—C11—C9	116.7 (3)	C26—C27—H27A	108.8
C12—C11—H11A	108.1	N6—C27—H27B	108.8
C9—C11—H11A	108.1	C26—C27—H27B	108.8
C12—C11—H11B	108.1	H27A—C27—H27B	107.7
C9—C11—H11B	108.1	N6—C28—N8	121.3 (3)
H11A—C11—H11B	107.3	N6—C28—N7	120.1 (3)
C13—C12—C11	114.1 (3)	N8—C28—N7	118.6 (3)
C13—C12—H12A	108.7	C8—N1—C9	125.7 (3)
C11—C12—H12A	108.7	C8—N1—H1	116 (2)
C13—C12—H12B	108.7	C9—N1—H1	118 (2)
C11—C12—H12B	108.7	C14—N2—C13	123.9 (3)
H12A—C12—H12B	107.6	C14—N2—H2	118.0
N2—C13—C12	111.3 (3)	C13—N2—H2	118.0
N2—C13—H13A	109.4	C14—N3—H3A	118 (3)
C12—C13—H13A	109.4	C14—N3—H3B	119 (2)
N2—C13—H13B	109.4	H3A—N3—H3B	122 (4)
C12—C13—H13B	109.4	C14—N4—H4A	118 (2)
H13A—C13—H13B	108.0	C14—N4—H4B	125 (3)
N2—C14—N4	121.7 (3)	H4A—N4—H4B	117 (3)
N2—C14—N3	119.8 (3)	C22—N5—C23	124.8 (3)
N4—C14—N3	118.4 (3)	C22—N5—H5	117 (2)
O5—C15—H15A	109.5	C23—N5—H5	118 (2)
O5—C15—H15B	109.5	C28—N6—C27	123.2 (3)
H15A—C15—H15B	109.5	C28—N6—H6	118.4
O5—C15—H15C	109.5	C27—N6—H6	118.4
H15A—C15—H15C	109.5	C28—N7—H7A	115 (2)
H15B—C15—H15C	109.5	C28—N7—H7B	115 (2)
O5—C16—C21	124.5 (4)	H7A—N7—H7B	127 (3)
O5—C16—C17	114.1 (3)	C28—N8—H8A	121 (2)
C21—C16—C17	121.4 (3)	C28—N8—H8B	120 (2)
C18—C17—C16	119.6 (3)	H8A—N8—H8B	118 (3)
C18—C17—H17	120.2	C2—O1—C1	118.2 (3)
C16—C17—H17	120.2	C16—O5—C15	118.8 (3)
C17—C18—C19	121.2 (3)	H1W—O1W—H2W	110.4 (17)
C17—C18—H18	119.4		
			
O1—C2—C3—C4	−178.2 (3)	C18—C19—C20—C21	2.6 (5)
C7—C2—C3—C4	2.5 (5)	O5—C16—C21—C20	179.4 (4)
C2—C3—C4—C5	−1.2 (5)	C17—C16—C21—C20	−1.6 (6)
C3—C4—C5—C8	179.9 (3)	O6—C20—C21—C16	179.2 (4)
C3—C4—C5—C6	−1.0 (5)	C19—C20—C21—C16	−1.0 (6)
C8—C5—C6—O2	1.2 (5)	C20—C19—C22—N5	0.0 (5)
C4—C5—C6—O2	−177.9 (3)	C18—C19—C22—N5	176.9 (3)
C8—C5—C6—C7	−178.9 (3)	N5—C23—C24—O8	−18.7 (4)
C4—C5—C6—C7	2.0 (4)	C25—C23—C24—O8	102.2 (4)
O1—C2—C7—C6	179.3 (3)	N5—C23—C24—O7	161.9 (3)
C3—C2—C7—C6	−1.5 (5)	C25—C23—C24—O7	−77.3 (4)
O2—C6—C7—C2	179.1 (3)	N5—C23—C25—C26	−178.7 (3)
C5—C6—C7—C2	−0.8 (5)	C24—C23—C25—C26	59.7 (4)
C4—C5—C8—N1	178.1 (3)	C23—C25—C26—C27	57.0 (4)
C6—C5—C8—N1	−1.1 (5)	C25—C26—C27—N6	−179.2 (3)
N1—C9—C10—O4	167.8 (3)	C5—C8—N1—C9	−174.4 (3)
C11—C9—C10—O4	−71.5 (4)	C11—C9—N1—C8	60.9 (4)
N1—C9—C10—O3	−14.6 (4)	C10—C9—N1—C8	−176.2 (3)
C11—C9—C10—O3	106.2 (4)	N4—C14—N2—C13	6.0 (5)
N1—C9—C11—C12	178.7 (3)	N3—C14—N2—C13	−175.9 (3)
C10—C9—C11—C12	57.7 (4)	C12—C13—N2—C14	79.9 (4)
C9—C11—C12—C13	55.3 (4)	C19—C22—N5—C23	−177.9 (3)
C11—C12—C13—N2	−179.3 (3)	C24—C23—N5—C22	−155.8 (3)
O5—C16—C17—C18	−178.4 (3)	C25—C23—N5—C22	80.4 (4)
C21—C16—C17—C18	2.5 (6)	N8—C28—N6—C27	−1.6 (6)
C16—C17—C18—C19	−0.8 (6)	N7—C28—N6—C27	178.4 (3)
C17—C18—C19—C22	−178.7 (3)	C26—C27—N6—C28	83.0 (5)
C17—C18—C19—C20	−1.8 (5)	C7—C2—O1—C1	−11.4 (5)
C22—C19—C20—O6	−0.7 (5)	C3—C2—O1—C1	169.3 (3)
C18—C19—C20—O6	−177.6 (4)	C21—C16—O5—C15	−9.9 (6)
C22—C19—C20—C21	179.5 (3)	C17—C16—O5—C15	171.0 (4)

### Hydrogen-bond geometry (Å, º)

**Table:**

*D*—H···*A*	*D*—H	H···*A*	*D*···*A*	*D*—H···*A*
N1—H1···O2	0.89 (1)	1.94 (3)	2.638 (4)	134 (3)
N4—H4*A*···O4	0.90 (1)	2.06 (1)	2.935 (4)	165 (3)
N5—H5···O6	0.90 (1)	1.90 (3)	2.600 (4)	134 (3)
N8—H8*A*···O7	0.90 (1)	2.03 (1)	2.914 (4)	166 (3)
N2—H2···O2^i^	0.86	1.92	2.758 (4)	166
N3—H3*A*···O3^i^	0.89 (1)	2.58 (3)	3.333 (4)	142 (3)
N3—H3*B*···O4^ii^	0.89 (1)	1.93 (1)	2.817 (4)	175 (4)
N4—H4*B*···O3^ii^	0.89 (1)	2.03 (1)	2.912 (4)	171 (3)
N6—H6···O6^iii^	0.86	1.89	2.705 (4)	158
N7—H7*A*···O8^iii^	0.89 (1)	2.50 (3)	3.202 (4)	135 (3)
N7—H7*B*···O7^iv^	0.90 (1)	1.91 (1)	2.800 (4)	173 (3)
N8—H8*B*···O8^iv^	0.89 (1)	2.05 (2)	2.911 (4)	161 (3)
O1*W*—H1*W*···O2^v^	0.94 (1)	2.33 (6)	2.882 (5)	117 (4)
O1*W*—H2*W*···O3^v^	0.94 (1)	1.98 (2)	2.881 (5)	159 (6)
C15—H15*C*···O1*W*^i^	0.96	2.56	3.451 (7)	155
C22—H22···O1*W*	0.93	2.53	3.359 (6)	149
C1—H1*C*···*Cg*1^vi^	0.96	2.96	3.669 (4)	132
C15—H15*C*···*Cg*2^vii^	0.96	2.98	3.762 (5)	139

Symmetry codes: (i) *x*, *y*−1, *z*; (ii) −*x*, *y*−1/2, −*z*; (iii) *x*, *y*+1, *z*; (iv) −*x*+1, *y*+1/2, −*z*+1; (v) *x*+1, *y*, *z*; (vi) −*x*, *y*−1/2, −*z*+1; (vii) −*x*+1, *y*+1/2, −*z*.
